# EVALUATION OF VGC ANALYZER BY COMPARISON WITH GOLD STANDARD ROC SOFTWARE AND ANALYSIS OF SIMULATED VISUAL GRADING DATA

**DOI:** 10.1093/rpd/ncab066

**Published:** 2021-05-03

**Authors:** Jonny Hansson, Lars Gunnar Månsson, Magnus Båth

**Affiliations:** Department of Biomedical Engineering, Hospital of Halland, Varberg SE-432 81, Sweden; Department of Radiation Physics, Institute of Clinical Sciences, The Sahlgrenska Academy at University of Gothenburg, Gothenburg SE-413 45, Sweden; Department of Radiation Physics, Institute of Clinical Sciences, The Sahlgrenska Academy at University of Gothenburg, Gothenburg SE-413 45, Sweden; Department of Medical Physics and Biomedical Engineering, Sahlgrenska University Hospital, Gothenburg SE-413 45, Sweden; Department of Radiation Physics, Institute of Clinical Sciences, The Sahlgrenska Academy at University of Gothenburg, Gothenburg SE-413 45, Sweden; Department of Medical Physics and Biomedical Engineering, Sahlgrenska University Hospital, Gothenburg SE-413 45, Sweden

## Abstract

The purpose of the present work was to evaluate the use of resampling statistical methods for analysis of visual grading data—implemented in the software VGC Analyzer—by comparing the reanalyzed results from previously performed visual grading studies with the results calculated by gold standard receiver operating characteristic (ROC) methodology, Obuchowski-Rockette (OR)-Dorfman–Berbaum–Metz (DBM) multiple-readers and multiple-case (MRMC) and by analysis of simulated visual grading data where the true distribution was presumed to be known. The reanalysis was performed on two multiple-reader studies with non-paired data and paired data, respectively. The simulation study was performed by simulating a large number of visual grading characteristics (VGC) studies and by analyzing the statistical distribution of null hypothesis (H_0_) rejection rate. The comparison with OR-DBM MRMC showed good agreement when analyzing non-paired data for both fixed-reader and random-reader settings for the calculated area under the curve values and the confidence intervals (CIs). For paired data analysis, VGC Analyzer showed significantly lower CIs compared with the ROC software. This effect was also illustrated by the simulation study, where the VGC Analyzer, in general, showed good accuracy for simulated studies with stable statistical basis. For simulated studies with unstable statistics, the accuracy in the H_0_ rejection rate decreased. The present study has shown that resampling methodology can be used to accurately perform the statistical analysis of a VGC study, although the resampling technique used makes the method sensitive to small data sets.

## INTRODUCTION

Visual grading characteristics (VGC) analysis, introduced by Båth and Månsson in 2007^(1)^, is a non-parametric rank-invariant method for comparing visual grading data from two imaging conditions. The method was primarily developed for use in the optimization of diagnostic radiology examinations, where the visual grading methodology is a common choice by e.g. medical physicists, radiologists and radiographers when planning to perform time-effective optimization projects in clinical environments. In VGC analysis, rating data (e.g. image quality ratings) for the two conditions (a reference condition and a test condition) are compared by producing a VGC curve, similar to how the rating data for normal and abnormal cases in receiver operating characteristic (ROC) analysis are used to create an ROC curve. Hence, the VGC curve is a plot of the proportion of ratings above a certain threshold for the test condition against the same proportion for the reference condition as the threshold is changed. Thereby, the separation between the two rating distributions is characterized by the area under the VGC curve (AUC_VGC_) (0 ≤ AUC_VGC_ ≤ 1). A low AUC_VGC_ (<0.5) indicates that the reference condition is rated higher, whereas a high AUC_VGC_ (>0.5) indicates that the test condition is rated higher. An AUC_VGC_ of 0.5 indicates that the image quality for the two conditions—on average—is rated the same.

After its introduction, VGC analysis has been recognized as a useful method in visual grading studies^(2–8)^, although the statistical analysis (the determination of the AUC_VGC_ and its associated uncertainty) is not straightforward. This deficiency has also been pointed out by other researchers^(9–10)^. From its resemblance to ROC data, it was initially proposed to make use of the existing ROC software for statistical analysis also of VGC data^(1, 11–13)^. However, ROC studies are almost exclusively based on independence between the normal and abnormal data sets, and to the best of the authors’ knowledge, this independence is assumed in all contemporary ROC software. By contrast, in VGC studies, it is common that there is a dependency between the two sets of rating data for the two compared conditions, e.g. data resulting from the same group of patients examined with two types of equipment. Furthermore, when evaluating two imaging conditions, a fundamental difference in the properties of an ROC study, compared with a VGC study, is that in ROC the statistical analysis is focused on the uncertainty of the difference between the two ROC curves originating from the two conditions, whereas in VGC, the analysis is focused on the uncertainty of the single VGC curve originating from the two conditions. As a consequence, the use of paired data for increased statistical power in a study in ROC is handled between two ROC curves^(14, 15)^, whereas in VGC, paired data are handled within one VGC curve.

As an aid for the analysis of VGC data, a non-parametric resampling methodology dedicated to the statistical analysis of VGC data was recently introduced by Båth and Hansson^(16)^. The method handles single- and multiple-readers, paired and non-paired data and calculates the VGC curve by the trapezoid rule as well as by the binormal curve fitting method. Since observers may use the rating scale differently, the AUC_VGC_ is calculated individually for each observer and is averaged to maintain the rank-invariant property of VGC. Based on the resampling techniques, bootstrapping and permutation, the 95% confidence interval (CI) of the AUC_VGC_ and the *p*-value are determined non-parametrically for testing the null hypothesis that the two compared conditions are equal (H_0_: AUC_VGC_ = 0.5). In the multiple-readers setting, the uncertainty of the area under the curve (AUC) (and hence the CI and the *p*-value) is determined by treating the observers either as a fixed effect (the uncertainty limited to the participating observers) or as a random effect (the uncertainty generalized to the population of observers). The method is implemented in the freeware VGC Analyzer, which is available by contacting the authors.

Despite its recent introduction, VGC Analyzer has already been used in several visual grading studies^(17–23)^. Also, the introduction of VGC Analyzer was accompanied by a study^(24)^ where the method was used on data from four previously published VGC studies to compare evaluations using VGC Analyzer with ROC software. However, in that study the VGC data were analyzed using now outdated ROC software. Furthermore, a thorough evaluation of the validity of the methods used in VGC Analyzer has not yet been performed. Therefore, a purpose of the present study was to compare VGC Analyzer with gold standard ROC software on visual grading data, where it can be assumed that the ROC methodology provides valid results. Additionally, since the validity of an evaluation based on a comparison between two methods that do not have access to the absolute truth is limited, a second purpose of the present study was to extend the evaluation of the methodology by performing a simulation study where the truth is known. By creating a large number of simulated visual grading studies and by performing statistical analysis on each study, the distribution of the results can be compared with the real and known distribution of the simulated studies.

## MATERIALS AND METHODS

### Statistical analysis by resampling

The two resampling methods used in VGC Analyzer are bootstrapping and permutation. In bootstrapping, collected data are reused by stochastically picking one sample at a time (with replacement) from the data sets to build new pseudo data sets. The number of pseudo data sets that can be built is nominally *n^n^*, where *n* is the number of samples in the original data set. However, the number of unique resampled data sets that can be obtained will be reduced because the order of the resampled data is irrelevant. Also, in image perception studies, the number of rating scale steps used is limited and hence the collected rating values can appear more than once. Assuming that collected samples of observers and cases are good representatives of their populations, a thorough bootstrap of the data will present the required information for the statistical evaluation of the study with no assumption needed of the underlying distribution^(25–28)^. The bootstrapping procedure in VGC Analyzer is used to calculate the CI of the achieved AUC_VGC_ (estimated within the 2.5 and 97.5 percentiles of the bootstrapped AUC_VGC_ values), whereas permutation is used to calculate the *p*-value. The *p*-value (defined as the probability to achieve at least the detected difference between the compared conditions, given the null hypothesis, H_0_, is true) is correspondingly calculated by gathering data from the two conditions to one virtual population, and from this virtual population produce new permuted conditions. The variation of the permuted results is used to determine the probability to achieve the actual detected result by chance. The AUC_VGC_ is calculated individually for each observer by the trapezoid method as well as by binormal adaptation^(15)^. If, in the binormal situation, the resampled data rule out binormal adaptation, the AUC is instead calculated by the trapezoid method. In the fixed-reader situation, the individual AUCs are averaged for all observers, whereas in the random-reader situation, the observers are bootstrapped to analyze the reader variation. Bootstrapping on observers is performed in both CI and *p*-value calculations. The effect of the observer variability on the *p*-value is, in the random-reader situation, added to the permutation by bootstrapping which of the observers to include in each permutation sequence in the same way as in the bootstrapping for CI. If the original data are uncorrelated (unpaired), the permutation is performed over all cases. If the original data are correlated (paired), the permutation randomly selects which rating in each pair of ratings from the test and reference conditions that should be assigned to the permuted condition A and which should be assigned to the permuted condition B. The solution for estimating the uncertainties introduced by reader variation in a VGC study is the main argument to develop these resampling methods instead of using the Mann–Whitney or Wilcoxon signed rank tests that are traditionally used for the statistical analysis of ordinal data.

### Comparison with gold standard ROC methodology

The gold standard ROC software used for the evaluation of VGC Analyzer was Obuchowski-Rockette (OR)-Dorfman–Berbaum–Metz (DBM) MRMC (The Medical Image Perception Laboratory, The University of Iowa, Iowa City, Iowa, USA)^(29)^. The comparison between VGC Analyzer and OR-DBM MRMC in the analysis of VGC data was performed by reanalyzing two studies (also used in the previously published evaluation study^(24)^) focused on the optimal tube voltage for conventional urography (Zachrisson *et al.*^(30)^) and the effect of radiation dose reduction in chest radiography of premature neonates (Carlander *et al.*^(31)^). Both original studies contained multiple-readers data. The reanalyzed studies had originally used ROCFIT (C. E. Metz, University of Chicago, Chicago, IL, USA)^(32)^ for the calculation of CI. ROCFIT, being a single-reader analysis tool developed for the ROC analysis, forces the user to pool the observer data in order to fit the analysis chart, whereas gold standard ROC software, such as OR-DBM MRMC, handles the multiple-readers and multiple-case (MRMC) settings. The analysis in this study was performed using the software package OR-DBM MRMC 2.51, which was written by Stephen L. Hillis, Kevin M. Schartz and Kevin S. Berbaum based on the methods initially proposed by Berbaum, Dorfman and Metz^(33)^ and Obuchowski and Rockette^(34)^ and later unified and improved by Hillis and colleagues^(35–37)^.

In Zachrisson *et al*.^(30)^, the images for the two compared conditions were acquired from two different patient groups (independent or non-paired data), whereas in Carlander *et al*.^(31)^, the images for both conditions were acquired from the same group of patients (dependent or paired data). The comparison was performed by reanalyzing the data from the two studies and comparing the calculated CI by the two methods. In the reanalysis, VGC Analyzer (version 1.0.2) was configured for the analysis of either paired or non-paired data, depending on the type of data, whereas OR-DBM MRMC (version 2.51), adapted for ROC data, as described in the Introduction section, treats the data as non-paired. The coupling of the data pairs is therefore not maintained in the analysis, and it can be assumed that OR-DBM MRMC overestimates the CI of the AUC from the paired data study (Carlander *et al*.^(31)^). The uncertainty of the AUC_VGC_ was determined both for the actual observers (fixed-reader) and for the population of observers (random-reader). The AUC_VGC_ was determined from curve fitting by the trapezoidal rule, both for OR-DBM MRMC and VGC Analyzer. The OR-DBM MRMC software was set to use the Jackknifing resampling technique since the bootstrapping technique is currently not available in version 2.51.

### Simulations

A specially designed simulation software, built on the VGC Analyzer resampling methodology, was used to evaluate its validity by the analysis of simulated studies. In the simulations, the same resampling methodology as in VGC Analyzer was used, but the input and output routines were modified to suit the simulation procedures. The study was performed by analyzing the results from a large number of simulated VGC studies in which ratings were produced by random sampling from pre-defined distributions. For each simulated VGC study, the simulation software was used to determine the CI of the AUC_VGC_ and the *p*-value.

The resampling methods used in VGC Analyzer were evaluated by simulating VGC studies with the null hypothesis, H_0_, set true (the probability distributions for the two conditions set equal). A similar assessment strategy has been previously used for ROC analysis by Roe and Metz^(38)^, where simulated stochastically distributed ROC data (‘null case studies’) were used to test the DBM MRMC analysis method. The accuracy of the method for the calculation of CI was evaluated by testing if the resampling (bootstrapping and permutation) ‘erroneously’ indicated the significant difference between the two conditions (i.e. AUC_VGC_ = 0.5 was not included in the 95% CI) in the intended H_0_ rejection rate (5%) of studies (thereby performing Type I errors at an α-level of 0.05). Correspondingly, the accuracy of the method for the calculation of *p*-value was evaluated by testing if the resampling (permutation) ‘erroneously’ indicated the significant difference between the two conditions (i.e. *p* < 0.05) in the intended H_0_ rejection rate (5%) of studies.

A random sequence was applied to produce artificial visual grading ratings from the two simulated conditions, defined by various properties of the simulated observers’ probability distributions. The properties that were altered were the shape (uniform, normal or wedged), the number of scale steps and the statistical variation between cases as well as within and between observers. Different visual grading studies were simulated by varying the settings in the number of cases and observers, all with the prerequisite of the compared conditions being equal in terms of probability distributions. Paired data studies were generated by introducing a correlation between each pair of cases in the simulations of ratings for the two conditions.

In the single-reader situation, the ratings for the reference condition were randomized according to the selected distribution of ratings (e.g. normal distribution with mean 3 and standard deviation (SD) 1). In the unpaired single-reader situation, the ratings for the test condition were created in the same way as for the reference conditions (and thus independent of the specific ratings for the reference condition), whereas in the paired single-reader condition, the rating for each case in the test condition was drawn from a random distribution centered around the rating for the corresponding case in the reference condition.

Multiple-reader situations were simulated by introducing a randomized ‘dummy-observer’ rating, indicating the general impression among the observers for each case. The ratings for the ‘dummy-observer’ were created in the same ways as for the single-reader. The rating for each observer was then randomized from a normal distribution, centered around the ‘dummy-observer’ rating for the given case and with an observer-specific SD, constant for all cases. The observer-specific SD was randomized from another normal distribution with zero mean and SD, *σ*_obs_. The ratings of the ‘dummy-observer’ were not included in the simulated result.

For each combination of property values, 100 000 studies were simulated, separated in groups of 10 000 for the determination of the standard error. Each simulated study included a new set of observers and cases. The fraction of the resampling analysis results that indicated statistically significant difference (by CI and *p*-value) was recorded as the H_0_ rejection rate. In the resampling analysis, the number of bootstraps was varied from 200 to 20 000. The standard settings in the simulations were 2000 bootstraps and 2000 permutations.

## RESULTS

### Comparison with OR-DBM MRMC

**Figure 1:**
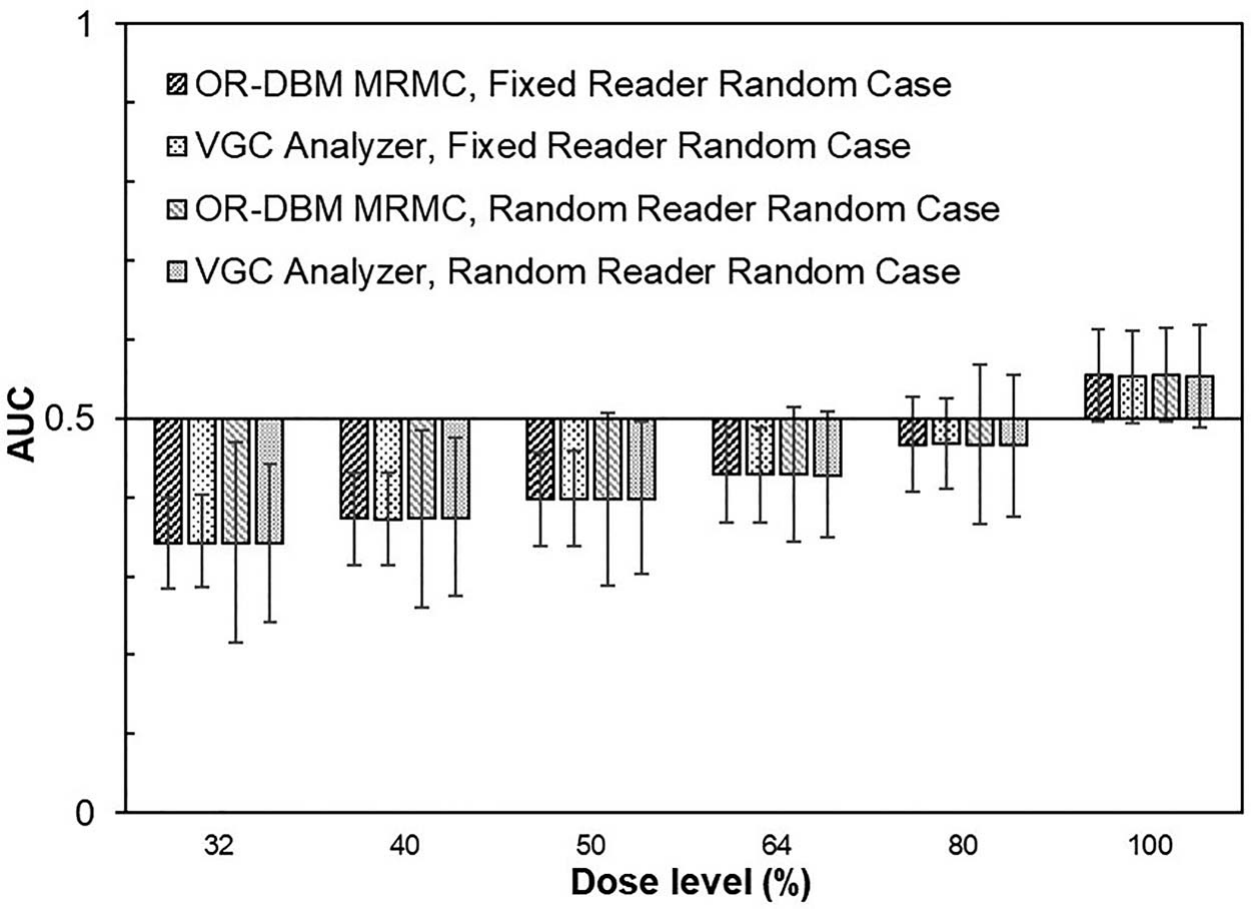
AUC and CI from Zachrisson *et al.*^(30)^ comparing two tube voltages for urography (55 kV at different dose levels vs. 73 kV at 100% dose) using five observers, three criteria and 31 cases per condition (non-paired data); in the analysis using OR-DBM MRMC and VGC Analyzer, criteria were pooled but the observers were treated either as fixed or random and the cases were treated as random; error bars are the 95% CIs given by the analysis software.

**Figure 2:**
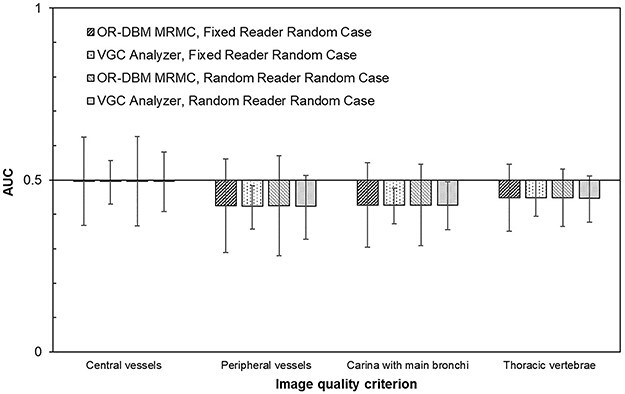
AUC and CI from Carlander *et al.*^(31)^ comparing two dose levels for neonatal chest imaging (80 vs. 100%) using five observers, four criteria and 24 cases per condition (paired data); in the analysis using VGC Analyzer, the paired data effect was taken into account, whereas in the analysis using OR-DBM MRMC, ROC data are presumed, and paired data effect is not taken into account in the analysis; observers were treated either as fixed or random and the cases were treated as random; error bars are the 95% CIs given by the analysis software.

The result of the comparison between VGC Analyzer and OR-DBM MRMC is presented in Figures 1 and 2, where the obtained AUC values and their 95% CIs for the reanalyzed studies are shown. It is important to note that the presented outcomes originate from identical data and that the differences are caused solely by the different methods of statistical analysis.

Applied to the non-paired data (Figure 1), the comparison showed almost identical results for AUC and for CI when the data were analyzed for the fixed-reader setting. The maximum difference in AUC was 0.2% and the mean difference in the CI was −1% (range: −4%, +1%) using OR-DBM MRMC as reference. When the data were analyzed for the random-reader setting, the agreement in AUC was unaffected (maximum difference: −0.3%), whereas a larger deviation in the CI was found: the mean difference in the CI was −9% (range: −21%, +8%).

Applied to paired data (where it can be assumed that OR-DBM MRMC overestimates the CI of the AUC), the comparison showed, likewise, almost identical results for AUC calculation (Figure 2). The maximum difference in AUC was −0.3% for the fixed-reader setting and −0.4% for the random-reader setting. However, for the uncertainty estimation, the difference in the CI was on the average −33% for the random-reader setting (range: −41%, −20%) and −52% for the fixed-reader setting (range: −58%, −47%) using OR-DBM MRMC as a reference.

### Simulations

In addition to the results presented below and illustrated in figures, the simulations showed improved stability in the analysis for both CI and *p*-value when the number of resamplings of the VGC data was increased. A small number of resamplings (200) increased the risk of misinterpreting the probability of significant differences in the simulated studies, whereas a higher number of resamplings (20 000) only resulted in minor improvements compared with the standard setting of 2000.

**Figure 3:**
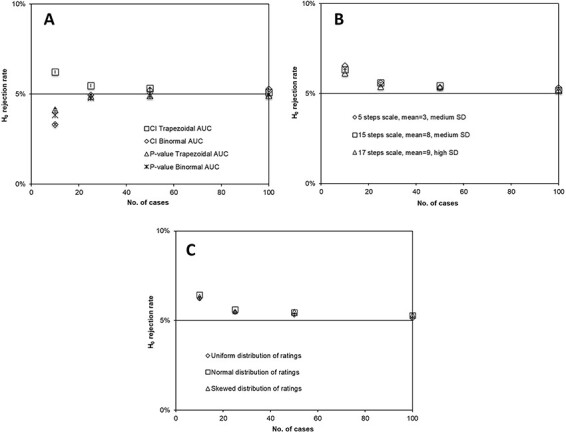
simulated data after 2000 bootstraps or permutations on 10 000 VGC studies with varying number of cases in a single-reader setting; error bars indicate the standard error from 10 consecutive simulation sessions of 10 000 VGC studies each; (**A**) H_0_ rejection rate from CI or *p*-value and AUC calculated with trapezoidal or binormal curve fitting for uniform distributions of ratings on a 5-steps scale (mean value = 3, SD = 1); (**B**) H_0_ rejection rate from CI and AUC calculated with trapezoidal curve fitting for normal distributions of ratings; The SD of the combined between-cases and within-observer variability was 1 scale step on a 5-steps scale, 1 scale step on a 15-steps scale (wider scale) and 4 scale steps on a 17 scale steps (finer scale), respectively; (**C**) H_0_ rejection rate from CI and AUC calculated with trapezoidal curve fitting for normal distributions of ratings with three different probability distributions; uniform, normal and skewed.

For large numbers of cases in the single-reader setting, the robustness was good for both trapezoid and binormal calculation of the AUC. However, for small numbers of cases, the trapezoid calculation led to a too high H_0_ rejection rate (resulting from an underestimated CI and leading to an increased risk of committing Type I errors), whereas the binormal curve fitting for the calculation of AUC led to a too low H_0_ rejection rate (resulting from an overestimated CI and an increased risk of committing Type II errors). This result was found for all combinations of settings and is exemplified in Figure 3a. When the *p*-value was used for hypothesis testing, a small number of cases in the AUC calculation led to a too low H_0_ rejection rate with both trapezoid and binormal curve fitting (Figure 3a). The number of scale steps and the width of the distribution of ratings had no evident effect on the H_0_ rejection rate **(**exemplified for the trapezoidal AUC in combination with CI in Figure 3b). Likewise, simulations of VGC studies with different rating distributions (uniform, normal and skewed) showed that the resampling methods were non-sensitive to the underlying probability distribution (Figure 3c).

**Figure 4:**
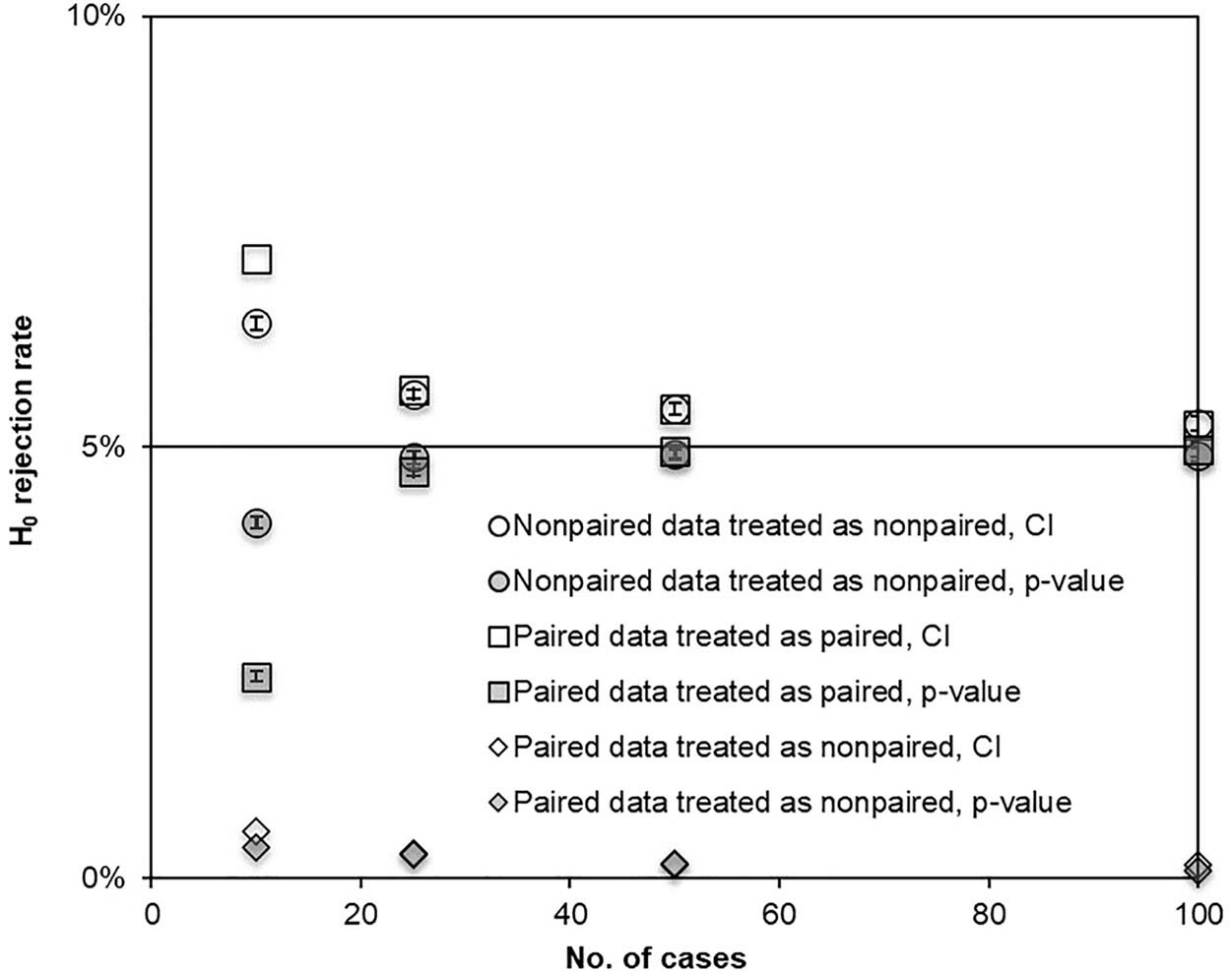
simulated data after 2000 bootstraps or permutations on 10 000 VGC studies with varying number of cases in a single-reader setting; simulated non-paired data and paired data treated as non-paired or paired; H_0_ rejection rate from CI or *p*-value and AUC calculated with trapezoidal curve fitting for normal distributions of ratings on a 5-steps scale (mean value = 3, SD = 1); error bars indicate the standard error from 10 consecutive simulation sessions of 10 000 VGC studies each.

Treating paired visual grading data as non-paired in the resampling processes resulted in an extremely low H_0_ rejection rate, visualized in Figure 4. For simulated single-reader settings, including 100 cases with normal-distributed ratings on a five-grade rating scale, the H_0_ rejection rate was only 0.1% for both *p*-value and CI evaluation. However, when paired data were treated as paired data in the resampling process, the H_0_ rejection rate was in agreement with non-paired data, although the dependency of the accuracy on the number of cases was slightly higher in the paired data situation.

**Figure 5:**
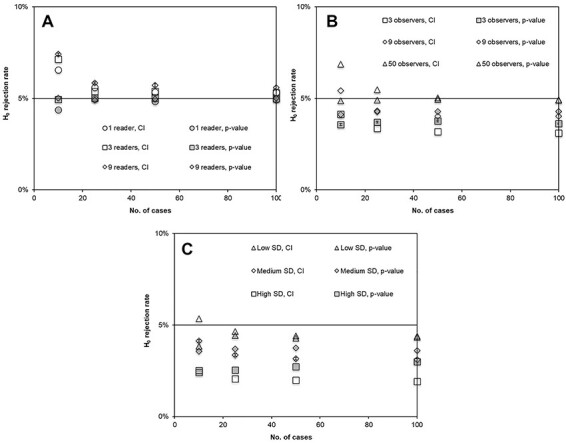
simulated data after 2000 bootstraps or permutations on 10 000 VGC studies with varying number of cases in multiple-readers settings; the ‘dummy-observer’ ratings were obtained from a normal distribution with mean value = 3 and SD = 1 on a 5-step rating scale; a value of *σ*_obs_ = 1 was used to create the multiple-reader situation; non-paired data treated as non-paired, normal distribution of ratings, AUC calculated with trapezoid curve fitting; error bars indicate the standard error from 10 consecutive simulation sessions of 10 000 VGC studies each; (**A**) H_0_ rejection rate from CI or *p*-value in studies where observers with medium variability are treated as fixed for two different set-ups of number of observers and compared with the single-reader setting; (**B**) H_0_ rejection rate from CI or *p*-value in studies where observers are generalized and treated as random for a large variation of observers with medium variability; (**C**) H_0_ rejection rate from CI or *p*-value in random-reader three-observer settings with low (×0.5), medium and high (×2) observer variability.

Introducing bootstrapping on multiple-readers studies showed no difference to single-reader studies as long as the data were analyzed for the actual observers (fixed-reader), independent of the number of observers included in the study (exemplified in Figure 5a). However, the simulations showed a too low H_0_ rejection rate when the analysis was set to be generalized to a population by resampling on observers (random-reader), and the error was largest for small numbers of observers, for both *p*-value and CI evaluation (exemplified in Figure 5b). With increasing number of observers, the H_0_ rejection rate for random-reader analysis approached the results for single-reader or fixed-reader analysis. Simulations of the dependency of H_0_ rejection rate on the observer variability showed an increased H_0_ rejection rate error with an increased observer variability (exemplified in Figure 5c).

## DISCUSSION

The purpose of the present study was to compare the resampling methods used by VGC Analyzer with state-of-the-art ROC methods and to perform a simulation study where the resampling analysis can be compared with a known truth. The study is limited by the small number of studies used for comparison with gold standard (valid for illustration purpose only) and the absence of comparable results from simulations using alternative methods for visual grading statistical analysis. The results of the performed study will be discussed in detail below, accompanied by practical recommendations for conducting VGC studies, based on the observations made in the present study.

The comparison between VGC analysis performed using VGC Analyzer and using ROC software (OR-DBM MRMC) was based on a reanalysis of data from previously published VGC studies, where ROCFIT was originally used for the analysis. Compared with ROCFIT, where the multiple-readers are treated as pooled, OR-DBM MRMC can handle the multiple-readers correctly. The reanalysis showed that the choice of software for the analysis did not affect the AUC_VGC_ value, whereas, for specific settings, the CIs differed. This discrepancy may affect whether the CI includes 0.5 or not and thus the determination of whether a statistically significant difference between the compared conditions has been found or not. The reasons for the discrepancy in the results obtained using the two types of software will be discussed in detail below.

As reported above, the multiple-readers reanalysis of the study presented in Figure 1 (non-paired data) showed that the use of VGC Analyzer resulted in similar CIs compared with OR-DBM MRMC when analyzing for the fixed-reader setting (−1%), whereas for the random-reader setting a discrepancy of −9% was measured, however, with a larger variation between the analyzed dose levels (range: −21%, +8%). The good agreement between the two analysis methods for fixed-reader analysis indicates the two methods to be reliable in this setting, whereas for multiple-readers, the larger variation in the CI is an indication that statistical analysis with the ambition to generalize to the population will introduce an uncertainty that needs special attention in the assessment of the study result.

The use of paired data in a VGC study will increase the power of the study. However, as paired data are treated differently in the ROC compared with VGC, the OR-DBM MRMC analysis used in the study by Carlander *et al.*^(11)^ (Figure 2) did not take the paired data into account in determining the CIs. The reported CIs were thereby broader compared with the results obtained using VGC Analyzer. This observation indicates that performing a statistical analysis on the visual grading data by the use of ROC-dedicated software, e.g. OR-DBM MRMC, for the analysis of data from paired VGC studies may lead to an underestimation of the significance of found differences and further leading to an increased risk of confirming H_0,_ although it is in fact not true (Type II errors). The broadening effect on CI width, when paired data handling is not included in the software, was actually higher with OR-DBM MRMC compared with ROCFIT in the previously performed study^(24)^, where the effect of pooling the observers by chance balanced the effect of not handling the paired data correctly.

The resampling methods used in the VGC Analyzer has in the present study been evaluated by performing statistical analysis (CI and *p*-value) of 100 000 sampled simulated studies to create each measurement point in the presented results. These simulations all had the setting of H_0_ being true, i.e. no difference between the compared conditions. Hence, significant differences found in the data arise from random variation and not from an actual difference. The premise was that in a distribution of simulated studies where the correct outcome is ‘no difference’ between the compared groups, only the proportion set by the study set-up, i.e. discrimination limits (e.g. 95% CI), will result in a correct H_0_ acceptance. On the other hand, the most extreme returns of the simulated studies will falsely be detected by the statistical analysis as a significant difference between the conditions, i.e. performing a Type I error. All evaluations performed in the simulation part of the present work is based on the premise that the ‘incapacity’ of the statistical analysis leading to an—actually false—rejection of H_0_ in the correct part of the individual studies of the simulated outcome is a good measure of the capability of the statistical analysis methods to discriminate significant differences. This strategy of detecting significant differences is supported by its similarity to the definition of *p*-value calculation, i.e. the probability of achieving a detected difference although H_0_ is true. Also, in simulated studies with sufficient sample size, i.e. stable statistical basis, the results from both CI and *p*-value calculations showed good agreement with the expected H_0_ rejection rate.

The resulting figure of merit from a visual grading study evaluated using VGC analysis is a single rank-invariant AUC_VGC_ value, produced by no parametric assumptions of the underlying rating distribution, although with an undefined uncertainty. In VGC Analyzer, the uncertainty is determined by measuring the contribution each given rating has to the resulting figure of merit by the use of non-parametric resampling. This enables a full conservation of the rank-invariant nature of VGC, with no assumptions of the underlying probability distribution in given ratings or resulting resampling data. The insusceptibility to limited expansion of the assessment scale, as shown in Figure 3b, is interpreted as a consequence of the rank-invariant nature of VGC. In contrast to traditional visual grading methods, where the ordinal scale in the given scores is transferred to a linear scale for statistical analysis, VGC makes no assumption of the ranking scale in which the scores are given. A concluding experience of this study is therefore that the set-up of the observers’ rating scale in a VGC study (and the following analysis using VGC Analyzer) needs little account taken to the subsequent statistical analysis and should instead be focused on the most clinically relevant scale setting for the observers.

Simulations of case and observer variability effects on H_0_ rejection rate showed increased underestimation of the significant difference probability with increased variability (Figure 5c). The simulation test cannot be directly transformed to a separated test of case, inter-reader or intra-reader variability, but in practice, the result emphasizes the importance of the observer education in order to minimize the study variations due to poor preparation. Not only will the statistical basis for the evaluation be weak, but the non-parametric statistical analysis used in the VGC Analyzer will also be weaker in its discrimination, thereby reducing the probability of detecting significant differences.

Resampling analysis of simulated multiple-readers studies without bootstrapping on observers (fixed-reader) showed a similar result to a single-reader study, whereas, when generalizing by bootstrapping on observers, the accuracy in the bootstrapping decreased. The accuracy decreased even more with an increased observer variability, i.e. significance testing by CI or *p*-value failed increasingly to reject H_0_ in studies that had an outcome with an ‘erroneously’ significant difference between the compared conditions, and hence, H_0_ should have been rejected. This underestimation of H_0_ rejection rate is interpreted as an effect of excessed broadening of the probability distribution (CI) by the bootstrapping when the underlying distribution is diffuse (also noted in the comparison with OR-DBM MRMC). The effect has been noticed by Efron^(28)^ and is expressed as the ‘dilation phenomenon’ in the bootstrap distribution—compared with the overall probability distribution—when the statistical variability of the observers’ ratings is increased. Dorfman *et al*.^(39)^ made the same conclusion when comparing statistical parameters from bootstrapping with those from the more established DBM method on ROC data from comparison of two examination procedures in neonatal radiology. When generalizing the ratings by bootstrapping both cases and observers in the study with only four observers (although, all four very specialized pediatric radiologists), bootstrapping showed a broader CI compared with DBM, whereas, when analyzing for fixed-reader, the results were comparable. A description of the phenomenon has also been presented by Samuelson and Wagner^(40)^.

In general, performing studies on a material where the statistical basis is weak will produce weak statistical analysis results. The performed simulation tests show that the weakness can be even weaker by the statistical analysis. The most critical situation found was the generalization of a study to the population of observers in studies, including few observers and/or large variation between observers. A non-surprising conclusion of this observation—also expressed by other researchers in the field^(38, 40, 41)^—is that a study should only be generalized to a population if the observers used in the study are statistically stable (i.e. give rise to low variation of the AUC_VGC_ sample mean and are good representatives of the population). Hence, for a human observer study intended to be generalized to the population of observers, a larger group of observers should be engaged in the study, and they should be as representative as possible for the population. If they are either too few or not representative, it is better to handle them as fixed-reader and to present the result as a fixed-reader result. This will increase the probability of achieving significant differences to the price of generalization validity, an experience supported also from similar research fields^(42)^.

Treating paired data as non-paired in the resampling analysis resulted in a substantial underestimation of significant differences between compared conditions (Figure 4). In a paired data study, where the same set of study objects is used on both conditions, the variation in ratings will mainly depend on the difference in ability between the conditions. This strong connection in the ratings will increase the power of the study set-up, which—if not used in the analysis—will be weakened with an underestimation of the statistical difference as a consequence. This experience is very important to bear in mind when performing a paired data study where cases are censored between first and second events, i.e. a planned paired data study must not be transformed into a non-paired data study. Therefore, when planning a study where paired data are possible (which is desirable), the risk of patients not being able to participate in both studies should be considered in the sizing of the study.

In the comparison of reanalyzed paired data—a situation where an ROC-intended software as OR-DBM MRMC is not adapted for correct handling and therefore cannot be considered as a gold standard—the results showed an increased CI compared with the VGC Analyzer of 40% for the random-reader setting (Figure 2). If—as an example for comparison—assuming normal distribution, a separation criterion for significant difference of 95% probability means that, by the use of the standard normal (*Z*) table, the CI is 1.96 times the SD, and if the CI is increased by 40%, the corresponding *Z* table value of 2.74 will result (from the *Z* table) in an increase of the separation criteria from 95 to 99.4%—or likewise in a H_0_ rejection rate decrease from 5 down to 0.6%. The corresponding H_0_ rejection rate for the fixed-reader setting, where the CI was 100% wider compared with the VGC Analyzer, will decrease to 0.01%. Simultaneously, the simulation study showed that H_0_ rejection rate decreased to 0.1% when paired data were treated as non-paired, as illustrated in Figure 4. Thus, concerning the handling of paired data, it can be concluded that the discrepancies between the VGC Analyzer and OR-DBM MRMC noted in the reanalysis of real data is confirmed in the simulation study.

Comparing the evaluations by CI with the *p*-value calculations showed good agreement for the simulated studies, given a sufficient sample size (Figures 3a and 5b). Also, the tendency of decreased accuracy for generalized multiple-readers studies, including few observers, were similar for the two methods (Figure 5b and c). However, in simulated settings with more stable statistics (i.e. single-reader, fixed-reader or random-reader with large numbers of observers), the single-reader settings showed the two methods to diverge in opposite direction from the ideal 0.05 level for simulated studies, including few cases (Figures 3a, 5a and b), with an enhanced effect for paired data studies (Figure 4). On the other hand, in the multiple-readers, setting the *p*-value calculation continued to show a correct H_0_ rejection rate even for a low number of cases (Figure 5a). For these settings, the bootstrapping showed a tendency to underestimate the CI (leading to a too high H_0_ rejection rate and a risk of committing Type I error). The reason for this divergence has not been thoroughly evaluated, although one possible source is the different resampling methods used in bootstrapping for CI and permutation for *p*-value. A suggested practical use of this effect in the analysis of results is that different results from CI and *p*-value calculations, in single-reader or fixed-reader settings, are indications of statistically unstable study set-ups. If, on the other hand, the two methods agree, the interpretation of the result is strengthened. As exemplified in Figure 5b and c, the adjacent action for the random-reader setting with few observers is to compare the random-reader results with the fixed-reader results, where a too large discrepancy between the analysis settings indicates a statistical instability within the observer group.

The original studies included in the previous evaluation study^(24)^ all used binormal curve fitting for the calculation of the AUC_VGC,_ whereas in the comparison with OR-DBM MRMC in the present study, the trapezoid rule was used. As previously described in this paper, both curve fitting methods have been shown to have both advantages and disadvantages in terms of accuracy in the AUC value, CI and *p*-value. The outcomes in the previous evaluation study illustrated this effect, where the use of binormal curve fitting resulted in larger deviation of AUC_VGC_ from 0.5 and wider CIs compared with the trapezoid rule. The resulting ability to discriminate the AUC_VGC_ from 0.5 with the two types of curve fitting was, however, not possible to conclude in the reanalysis study performed in this work, as the truth was not known. In the simulation study, the use of binormal curve fitting for calculation of AUC showed, especially in studies including few cases, the method to be sensitive to statistically extreme outcomes. When few ratings are included, the probability of the binormal curve fitting algorithm to fail cannot be neglected. When this failure is detected, the VGC Analyzer will switch to trapezoid calculation. As the AUC value calculated by this method—in reference to AUC = 0.5—is in general underestimated compared with the calculation by binormal curve fitting, the use of two different methods for AUC calculation will in the end introduce a systematic broadening of the non-parametric AUC distribution. As the validity of the statistical analysis is dependent on the stability in the resampled AUC values and not on the absolute AUC value, this broadening of AUC distribution will result in a decreased detection probability of significant differences, as shown in Figure 3a. Furthermore, the binormal curve fitting method assumes that the distribution of ratings given can be fitted to two normal distributions on the same scale. According to Hanley^(43)^, any distribution can be fitted to be normal distributed by scaling. However, if the two compared distributions need different scalings to be normally distributed, some form of compromise is needed. Also, practical experience has shown that given ratings from an image perception study will not always form a monotonic slope of the ROC curve and, in order to achieve credible AUC values, adjustments of data may be needed^(44, 45)^. Accordingly, as one aim of the VGC Analyzer is to present a reliable method with no assumption of the underlying distribution in the collected data, the trapezoid method was mainly used for the calculation of the AUC in the present evaluation of the software. For practical reasons, as previously described, the trapezoid method was also used in the comparison between VGC Analyzer and OR-DBM MRMC.

## CONCLUSIONS

In the present study, VGC Analyzer has been compared with gold standard ROC methodology by reanalysis of visual grading data and is evaluated by performing simulation studies where the truth was assumed to be known. The reanalysis showed good agreement between VGC Analyzer and OR-DBM MRMC when analyzing non-paired data, especially for the fixed-reader setting. For the random-reader setting, the uncertainty increased for both analysis methods. When analyzing paired data, the VGC Analyzer indicated substantially smaller CI compared with the ROC data-intended OR-DBM MRMC, where a correct handling of paired visual grading data is not implemented. The effect of incorrect handling of paired data was verified by the simulation study. The simulation study also showed VGC Analyzer to give reliable results from studies with stable statistical basis and to be insusceptible to the assessment scale and distribution of ratings. However, for simulated studies where the number of cases or observers was low, the H_0_ rejection accuracy decreased.

The take-home message of this evaluation is thus that for VGC studies where the statistical basis is stable, the statistical analysis by resampling will give reliable results, whereas with an unstable statistical basis, the analysis will enhance the weakness of the study. In single-reader or fixed-reader settings, an indication of the statistical basis of the study can be found by comparing the analysis results given by the VGC Analyzer for the two analysis methods (CI and *p*-value): if agreement, the statistical basis is likely to be stable; if disagreement, the statistical basis is likely to be unstable. A generalization to the population of observers should always be avoided when the observers are few. Here, a large discrepancy between the analysis results from the random-reader setting and the fixed-reader setting can give an indication of a statistical instability within the observer group.
